# Tenotomy in Convergent Squint

**Published:** 1897-12

**Authors:** A. Bethune Patterson

**Affiliations:** Atlanta, Ga.


### TENOTOMY IN CONVERGENT SQUINT.

By A. BETHUNE PATTERSON, M.D.,

ATLANTA, GA.

The guiding muscles of the eyeball are the internal and external recti, the superior and inferior recti, and the obliques, and are the muscles involved in strabismus. There are several methods of treating strabismus which are based upon the theories entertained as to the cause, and which will not be taken into this discussion, as my principal object now is to call attention to the too frequent and uncientific operation of cutting the internal rectus muscle in convergent squint. The indications in cross-eye have always been to straighten the eye. Modern research has led us a step further and established a second indication, that of reestablishing the function of observing with the deviating eye, for binocular vision becomes suspended soon after parallelism of the visual lines is lost. Coincident with the deviation double vision makes its appearance, and usually of short duration, for the squinting eye soon learns to suppress its image. The fusion of the images when once suspended, is by no means easily restored ; it is a tedious and painstaking task to bring about a restoration of the physiological function, and should be of grave concern to the conscientious operator. It must not be understood that the straightening, or more properly the apparent straightening, has restored to the eye its former habit of seeing, but on the other hand when fusion takes place it is proof positive of the parallelism of the lines of vision. I hold that the treatment can only be regarded as successful when these several conditions are realized, for the eye is useless and virtually blind so long as the image is allowed to remain suppressed. Then it should be the principal object in the treatment or management of squint, to restore to the eye its physiological usefulness ; this becomes exceedingly difficult, and in many cases impossible. After cutting the internal rectus, the eye turns a little up and remains in this position. This hyperphoria becomes a grave complication and must be overcome before binocular vision is attained. It must be said that considering the strabismus operation from a cosmetic standpoint only is an obsolete custom, yet there are a few old operators who continue the practice of twenty years ago. It is to be regretted that this branch of eye surgery has been neglected by many of our distinguished operators. It is true that the simple tenotomy is the quickest and easiest way of disposing of these cases, coupled with the fact that the improvement in the appearance of the subject that follows is satisfactory to those who are ignorant of the true condition. In an article published in the *Southern Medical Record* six or seven years ago, I protested against the indiscriminate cutting of the internal rectus in convergent squint. The following I select from a number of cases illustrative of the evils of which this paper complains of operating wholly from a cosmetic standpoint:

Miss P. has been under my care for several years. She had her internal rectus muscle cut for convergent strabismus by a distinguished surgeon a short time after the squint appeared. The double vision had subsided only a few weeks. He was ignorant of the existing hypermetropia or far-sight which was three and a half diopters. When she came under my treatment there was still a slight convergence, with the usual turning up (eso-hyperphoria). I succeeded in reestablishing binocular vision by doing a graduated tenotomy and correcting the hypermetropia with glasses. I feel very confident that at the time of the first operation, if her hypermetropia had been corrected, and the accommodation kept quiet for some time by the instillation of atropin, the cutting of the muscle would not have been necessary. Very many cases of convergent strabismus in children are due to far-sight, and if taken in hand early can be cured by glasses which correct the refractive error. Then again there are a number of others due to weak external recti, which can be cured by developing the weak muscle. A complete severance of the muscle should only be made after a due consideration of all the conditions incident to strabismus.

